# Pathogenicity of germline *VHL* variants is associated with renal cell carcinoma size in von Hippel-Lindau disease

**DOI:** 10.20945/2359-4292-2024-0354

**Published:** 2025-01-31

**Authors:** Gustavo H. Mori, Gustavo F. C. Fagundes, Lucas S. Santana, Felipe Freitas-Castro, Ana Caroline F. Afonso, Delmar M. Lourenço Jr., Maria Adelaide A. Pereira, Fabio Y. Tanno, Victor Srougi, Jose L. Chambo, Mauricio D. Cordeiro, William C. Nahas, Ana O. Hoff, Maria Candida B. V. Fragoso, Berenice B. Mendonca, Ana Claudia Latronico, Madson Q. Almeida

**Affiliations:** 1 Unidade de Adrenal, Laboratório de Endocrinologia Molecular e Celular LIM25, Divisão de Endocrinologia e Metabologia, Hospital das Clínicas, Faculdade de Medicina da Universidade de São Paulo, São Paulo, SP Brasil; 2 Laboratório de Hormônios e Genética Molecular LIM42, Divisão de Endocrinologia e Metabologia, Hospital das Clínicas, Faculdade de Medicina da Universidade de São Paulo, São Paulo, SP Brasil; 3 Divisão de Urologia, Hospital das Clínicas & Instituto do Câncer do Estado de São Paulo (ICESP), Faculdade de Medicina da Universidade de São Paulo, São Paulo, SP Brasil; 4 Unidade de Oncologia Endócrina, Instituto do Câncer do Estado de São Paulo (ICESP), Faculdade de Medicina da Universidade de São Paulo, São Paulo, SP Brasil

**Keywords:** Von Hippel-Lindau disease, renal cell carcinoma, adrenal paraganglioma, genetics

## Abstract

**Objective:**

In this study, our aim was to search for new genotype-phenotype correlations in patients with Von Hippel-Lindau (VHL) disease.

**Subjects and methods:**

We retrospectively studied 53 consecutive patients with VHL disease and confirmed genetic diagnoses from 32 relatives.

**Results:**

Most *VHL* pathogenic or likely pathogenic variants were missense (18 out of 32; 56.25%). The median size of the large carcinoma (RCC) was 3.6 cm (interquartile range, 2.8 to 6.5 cm). Interestingly, the size of the large RCC in patients harboring *VHL* pathogenic variants (n = 9) was significantly greater than that in patients with *VHL* likely pathogenic (n = 7) variants (5.4 cm [3.65 to 6.6] vs. 2.9 cm [2.45 to 3.35]; *p* = 0.008). Moreover, adrenal paraganglioma (PGL) (82.35% vs. 17.65%; *p* = 0.0001) and pancreatic neuroendocrine tumor (PNET) (81.81% vs. 18.18%; *p* = 0.007) were associated with missense *VHL* pathogenic or likely pathogenic variants compared with non-missense defects. In contrast, central nervous system (CNS) hemangioblastomas (HBs) (90.47% vs. 53.12%; *p* = 0.004), pancreatic cysts (76.19% vs. 28.12%; *p* = 0.001) and RCCs (57.14% vs. 12.5; *p* = 0.001) were more common in patients with non-missense *VHL* variants.

**Conclusion:**

VHL pathogenic variants were associated with larger RCCs than were *VHL* likely pathogenic variants.

## INTRODUCTION

Von Hippel-Lindau (VHL) disease is a hereditary autosomal-dominant neoplasia syndrome characterized by the development of benign and malignant tumors in multiple organ systems, including retinal angiomas and central nervous system (CNS) hemangioblastomas (HBs), renal cell carcinomas (RCCs), renal cysts, adrenal paragangliomas (PGLs), pancreatic cysts, pancreatic neuroendocrine tumors (PNETs), endolymphatic sac tumors and epididymal or broad ligament cystadenomas (^1^). VHL disease is caused by germline defects in the *VHL* tumor suppressor gene, which is located on chromosome 3p25-p26 and has three exons (^2^). Loss of heterozygosity is found in half of VHL-related tumors (^3^). The incidence of VHL disease ranges from 1 in 36,000 to 45,000 live births, and its prevalence is estimated to be between 1 in 38,000 and 91,000 individuals. Approximately 20% of *VHL* disease cases result from a *de novo* mutation and do not have a family history (^4^).

A germline pathogenic or likely pathogenic variant in *VHL* confirms the diagnosis of VHL (^5^). The clinical criteria for the diagnosis of VHL disease include one or more VHL-related tumors (*e.g.,* CNS HB, retinal angiomas, adrenal PGL, RCC, or endolymphatic sac tumors) and a family history of VHL disease; those without a family history must have two VHL-related tumors (^6^). VHL disease can be classified into type 1 or type 2 according to the absence or presence of adrenal PGL, respectively (^7^,^8^). Most *VHL* defects in patients with VHL disease type 1 are microdeletions/insertions, nonsense mutations, or deletions, whereas VHL disease type 2 is characterized by missense *VHL* pathogenic or likely pathogenic variants (^9^,^10^,^11^). In this study, our aim was to search for new genotype-phenotype correlations in patients with VHL disease.

## SUBJECTS AND METHODS

This study was approved by the Ethics Committee of the Clinics Hospital (#06194919.1.0000.0068) and the Cancer Institute of Sao Paulo State (#1448/19), University of São Paulo Medical School. A written informed consent form was signed by all patients or caregivers. We retrospectively studied 53 consecutive patients with VHL disease referred to the Division of Endocrinology at our institution. Since 2000, we have offered genetic testing to all patients with a clinical diagnosis of VHL and their first-degree relatives. We searched for VHL manifestations at diagnosis and during the follow-up. Unfortunately, patients with VHL disease are not referred early to specialized centers after the first tumor diagnosis. Thus, most patients have VHL tumors at the time of diagnosis. After the patients were referred to our institution, clinical and imaging follow-up was performed according to the surveillance guidelines proposed by Nielsen and cols. (^1^). Biochemical and imaging diagnoses of adrenal and extra-adrenal PGLs followed the Endocrine Society guideline recommendations (^12^). The measurement of RCC was performed at diagnosis, using the largest diameter of the largest lesion before any intervention.

Germline DNA was extracted from peripheral blood using the salting-out method. The genetic investigation was initially performed in germline samples using automated Sanger sequencing of VHL coding regions, as previously described (^13^). The following oligonucleotides were used: exon 1, forward *5’-CTAGCCTCGCCTCCGTTAC-3’* and reverse *5’-GTCACCCTGGATGTGTCCTG-3’;* exon 2, forward *S’-TTAGCCAGGACGGTCTTGAT-3’* and reverse *5’-CGTACAAATACATCACTTCCATT-3’;* and exon 3, forward *5’-TACTACAGAGGCATGAACACC-3’* and reverse *5’-CCCCTAAACATCACAATGC-3’.*

Multiplex ligation-dependent probe amplification (MLPA) for *VHL* was performed to investigate large deletions in PGL patients with clinical diagnosis of VHL and negative diagnosis after Sanger sequencing using the SALSA MLPA P016 *VHL* Probemix (MCR Holland) (^13^). The amplified fragments of MLPA were subjected to capillary electrophoresis in an ABI Prism 3130XL Genetic Analyzer (Thermo Fisher Scientific). The MLPA data were obtained using GeneMapper 5.0 (Thermo Fisher Scientific) and analyzed using Coffalyser software (MCR Holland).

Germline *VHL* variants were classified according to the American College of Medical Genetics and Genomics (ACMG) and the Association for Molecular Pathology (AMP) guidelines, including the most recent Clinical Genome Resource (ClinGen) Sequence Variant Interpretation Group recommendations (^14^,^15^).

### Statistical analysis

Descriptive statistics are reported as absolute (n) and relative frequencies (%) for qualitative variables and as medians with ranges and interquartile ranges (IQRs) for quantitative variables. Quantitative variables were compared between the groups using the Mann-Whitney U test. The normality assumption was evaluated using the Shapiro-Francia test. To assess the associations between two categorical variables, we used Pearson’s chi-square test or Fisher’s exact test. All hypotheses were two-sided and tested at a 5% significance level; a p value < 0.05 was considered statistically significant. Calculations were performed using the SPSS software (25.0; SPSS Inc., Chicago, IL, USA).

## RESULTS

A total of 53 patients (29 males and 24 females) with VHL disease from 32 relatives were evaluated. The median age at diagnosis was 20 years (IQR, 12 to 32.25 years). The median follow-up was 117 months (IQR, 71 to 209 months). Among the 53 patients, only nine patients were diagnosed with VHL disease by familial screening. CNS HB was the most common VHL-related tumor (67.92%), followed by adrenal PGL (64.15%), pancreatic cysts (47.17%), and PNET (41.51%) **(Table 1).**

**Table 1.** Tumor spectrum and genotype-phenotype correlations in 54 patients with von Hippel-Lindau (VHL) disease

Tumor	Frequency n (%)	Missense (n = 33)	Non-missense (n = 21)	p value
Pheochromocytoma	34 (62.96%)	28 (82.35%)	6 (17.65%)	0.000
Paraganglioma	7 (12.96%)	7 (100%)	0	0.022
Retinal angiomas	20 (37.03%)	9 (45%)	11 (55%)	0.075
CNS HB	36 (66.66%)	17 (47.22%)	19 (52.78%)	0.004
Pancreatic cysts	25 (46.29%)	9 (36%)	16 (64%)	0.001
PNET	22 (40.74%)	18 (81.82%)	4 (18.18%)	0.007
Renal cyst	21 (38.88%)	10 (47.62%)	11 (52.38%)	0.124
RCC	16 (29.62%)	4 (25%)	12 (75%)	0.001

CNS: central nervous system; HB: hemangioblastoma; PNET: pancreatic neuroendocrine tumor; RCC: renal cell carcinoma.

Bilateral adrenal PGL was diagnosed in 17 out of 34 (50%) patients. Seven of them (41.2%) were synchronous. The median age at diagnosis was 16 years (IQR, 11 to 27.5 years). The median large diameter was 4.0 cm (IQR, 2 to 6.65 cm). Single or multiple abdominal PGLs were identified in 7 patients, with a median large diameter of 3.10 cm (IQR, 1.7 to 4.3 cm). PNETs were identified in 22 out of 53 (41.51%) patients. The median age at diagnosis was 31 years (IQR, 21 to 44.75 years). The median large diameter of PNET was 2.1 cm (IQR, 1.15 to 4 cm). Multiple PNETs were diagnosed in 12 out of 22 patients (54.54%).

**Figure 1.**
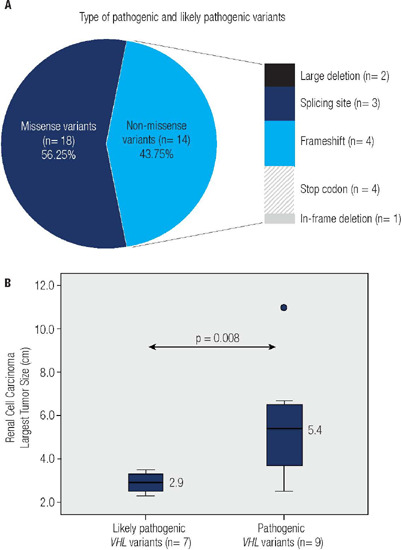
**(A)** Types of 32 distinct variants identified in 53 patients with VHL. **(B)** The pathogenicity of germline *VHL* variants, classified according to the American College of Medical Genetics (ACMG), is correlated with renal cell carcinoma tumor size.

Most *VHL* pathogenic or likely pathogenic variants were missense (18 out of 32 relatives; 56.25%) **(Figure 1A).** The remaining *VHL* pathogenic or likely pathogenic variants were as follows: stop codon (n = 4), frameshift (n = 4), splicing site (n = 3), large deletion (n = 2), and in-frame (n = 1). Among the *VHL* variants, 17 (53.12%) were located in exon 3, 9 (28.12%) in exon 1, and 3 (9.37%) in exon 2.

Next, we searched for new genotype-phenotype correlations in VHL disease. With respect to renal lesions, 21 out of 53 patients (39.62%) had renal cysts, and 16 (30.19%) had RCCs **(Table 1).** The median size of the large renal cyst was 2.1 cm (IQR, 1.32 to 3.6 cm). The median size of the large RCC for each patient was 3.6 cm (IQR, 2.8 to 6.5 cm). Interestingly, the size of the large RCC in patients harboring *VHL* pathogenic variants (n = 9) was significantly greater than that in patients with *VHL* likely pathogenic (n = 7) variants (5.4 cm [IQR, 3.65 to 6.6] vs. 2.9 cm [IQR, 2.45 to 3.35]; *p* = 0.008) **(Figure 1B).** The type of mutation was not associated with the pathogenicity of the variants in patients with RCC ([likely pathogenic, one missense, and six non-missense] vs. [pathogenic, three missense, and six non-missense], p = 0.383). Moreover, age at RCC diagnosis was not significantly different between patients with likely pathogenic and pathogenic variants ([45.5 years, IQR 32.5 to 49.25] vs. [46 years, IQR 29.25 to 51.5], p = 1.0). With respect to RCC diagnosis, 10 out of 14 patients had RCC at the time of diagnosis, and four out of 14 developed RCC during follow-up. Among the four patients with RCC diagnosed during the follow-up, two had *VHL* pathogenic variants (RCC diagnosis after 2 years and 16 years after VHL diagnosis), and two had likely pathogenic variants (RCC diagnosis after 10 years and 38 years after VHL diagnosis).

Moreover, missense *VHL* pathogenic or likely pathogenic variants were significantly associated with adrenal PGL (82.35% vs. 17.65%; p = 0.0001) and PNET (81.81% vs. 18.18%; p = 0.007) compared with non-missense defects **(Table 1).** In contrast, CNS HBs (90.47% vs. 53.12%; p = 0.004), pancreatic cysts (76.19% vs. 28.12%; p = 0.001), and RCCs (57.14% vs. 12.5; p = 0.001) were significantly more common in patients with non-missense *VHL* pathogenic or likely pathogenic variants.

None of the renal cell carcinoma (RCC) patients in our study had metastases or experienced disease-related deaths. Only two patients in our cohort died from VHL-related causes: one due to complications following neurosurgery to remove a hemangioblastoma, and the other from a locally advanced neuroendocrine pancreatic tumor.

## DISCUSSION

In our study, we demonstrated a new genotype-phenotype correlation in patients with VHL disease. VHL disease type 2 is caused mainly by germline missense *VHL* pathogenic or likely pathogenic variants and is characterized by the presence of adrenal PGL (1). Approximately 50% of adrenal PGLs in VHL disease are bilateral and are usually treated by cortical-sparing adrenalectomy (^16^). Moreover, PNETs are also associated with VHL disease type 2 (10,17). In contrast, CNS HBs and pancreatic cysts are more common in patients with VHL disease type 1 (^10^,^17^,^18^). Chiorean and cols. (^19^) conducted a systematic review and reanalyzed data on 634 unique VHL variants in 2,882 patients. Overall, the findings were consistent with previous descriptions of *VHL* genotype-phenotype correlations (^19^). We previously described some of the patients with VHL disease included in this study, but only eight patients with RCCs were included in the previous cohort (^17^). In agreement with previous findings (^10^), RCCs were more strongly associated with VHL disease type 1. In the present analysis, we explored the genotype-phenotype correlation involving RCCs in VHL disease. We demonstrated that the pathogenicity of the germline VHL variants was correlated with RCC size. Germline *VHL* pathogenic variants were associated with larger RCCs than were those from patients harboring *VHL* likely pathogenic variants.

Most of the germline *VHL* variants were located in exon 3, with codon 167 being a hotspot in our cohort. In a large multicentric study, PNETs occurred more frequently in patients with intragenic exon 3 defects than in those with defects in exons 1 and 2 (^20^). Furthermore, codons 161 and 167 in exon 3 were more strongly associated with adrenal PGL and PNET (^10^,^20^). Size (>2.8 cm) and exon 3 mutations (mostly in codons 161 and 167) confer an increased risk of malignancy in PNETs of patients with VHL disease (^20^,^21^). These findings underscore the importance of personalized management of patients with VHL disease according to mutational status.

*VHL* genetic alterations reduce VHL protein activity, which results in stabilization and subsequent constitutive activation of the transcription factor hypoxia-inducible factor 2α (HIF-2α), independent of oxygen concentrations (^22^). Recently, the HIF-2α inhibitor belzutifan was approved by the FDA for the treatment of RCC related to VHL disease (^23^). In a phase 2, open-label, single-group trial, belzutifan showed activity in patients with RCC and other neoplasms associated with VHL, such as PNET, pancreatic cysts, and HBs (^24^). After a median follow-up of 21.8 months, an objective response was observed in 49% of RCCs in patients with VHL disease (^24^).

Kwon and cols. (^25^) demonstrated that RCC size is an independent prognostic factor for overall survival in VHL disease patients. The 5-year overall survival rate was 85.6% for all patients, 96.9% for patients without RCC, 83.6% for patients with RCC < 3 cm, and 75.8% for patients with RCC ≥ 3 cm. In the context of this novel targeted therapy for VHL disease, our findings might be used in clinical practice to personalize treatment with belzutifan on the basis of the pathogenicity of germline *VHL* variants according to the ACMG classification. In other words, RCCs in patients with VHL disease caused by germline *VHL* pathogenic variants may benefit from preferential treatment with belzutifan due to the potential risk of tumor growth.

One strength of our study was the identification of a new genotype-phenotype correlation in a cohort of VHL patients from a single institution. However, limitations of our study were its retrospective design and relatively small number of patients compared with previous multicentric collaborative studies (^19^,^20^). Therefore, our findings need to be validated in a larger cohort of VHL patients to better delineate the relationship between pathogenicity classification and RCC size. The genetic variant classification by the ACMG/AMP is primarily based on population frequency, familial segregation, in silico predictions, and functional studies (^14^,^15^). Therefore, we hypothesize that the ACMG/AMP classification holds significant biological importance. Another limitation was the exclusion of VHL patients who were followed only by other specialties (gastrointestinal surgery, neurosurgery, ophthalmology, etc.) and lacked a genetic diagnosis. This may explain the high frequency of type 2 VHL disease observed in our cohort.

In conclusion, we demonstrated a new genotype-phenotype correlation in patients with VHL disease. Germline *VHL* pathogenic variants were associated with larger RCCs than were tumors from patients harboring germline *VHL* likely pathogenic variants.
